# Enlarged perivascular spaces in the basal ganglia region are associated with white matter interstitial fluid content via deep medullary vein dysfunction

**DOI:** 10.3389/fnhum.2025.1656036

**Published:** 2025-09-30

**Authors:** Haiyuan Lan, Xinjun Lei, Qingchun Wang, Lebing Wang, Kunwang Li

**Affiliations:** ^1^Department of Radiology, Lishui Hospital of Traditional Chinese Medicine Affiliated Zhejiang Chinese Medical University, Lishui, China; ^2^Department of Pain Management, Wenzhou Medical University Lishui Hospital, Lishui, China

**Keywords:** enlarged perivascular space, interstitial fluid, diffusion tensor imaging, free water, deep medullary vein

## Abstract

**Objective:**

To investigate the effect of enlarged perivascular spaces in the basal ganglia (BG-EPVS) on interstitial fluid (ISF) content in white matter and to assess the mediating role of deep medullary vein (DMV) dysfunction in this relationship among patients with cerebral small vessel disease (CSVD).

**Methods:**

Magnetic resonance imaging and clinical data were collected from 166 patients with CSVD. EPVS score (0–4) was evaluated on T2-weighted images on the basis of the number of EPVS in a single slice. DMV score (0–18) was assigned on susceptibility-weighted imaging according to signal continuity and clarity. Free water (FW) values, representing ISF content in white matter, were derived from diffusion tensor imaging.

**Results:**

The mean age of the participants was 63.6 ± 11.1 years. The median score was as follows: BG-EPVS, 1 (IQR: 1–2); centrum semiovale EPVS (CSO-EPVS), 1 (IQR: 1–2); CSVD score, 1 (IQR: 0–2), and DMV, 4 (IQR: 1–11). The mean FW values were 0.25 ± 0.02. No significant correlation was observed between CSO-EPVS and FW (*r* = 0.112, *p* = 0.119). In contrast, BG-EPVS showed a moderate positive correlation with both DMV score (*r* = 0.594, *p* < 0.001) and FW (*r* = 0.521, *p* < 0.001). DMV score was also moderate positively correlated with FW (*r* = 0.557, *p* < 0.001). These associations remained significant after adjusting for age, sex, and vascular risk factors (all *p* < 0.05). Mediation analysis confirmed that DMV score significantly mediated the relationship between BG-EPVS and FW, independent of covariates.

**Conclusion:**

Higher BG-EPVS score is significantly associated with increased ISF content in white matter, with DMV dysfunction serving as a key mediator of this association.

## 1 Introduction

White matter interstitial fluid (ISF) has recently emerged as a novel neuroimaging biomarker with significant relevance to neurodegenerative disorders. The accumulation of ISF has been shown to correlate positively with the severity of cerebral small vessel disease (CSVD) (Man et al., 2024) and is also strongly associated with neurocognitive impairment and the pathogenesis of Alzheimer’s disease (Dumont et al., 2019; Li et al., 2024). However, the underlying pathophysiological mechanisms responsible for elevated ISF levels remain poorly understood.

Enlarged perivascular spaces (EPVS), a key imaging marker of CSVD, are predominantly located in the basal ganglia (BG) and centrum semiovale (CSO). Recent studies have reported a positive association between high-grade EPVS and increased ISF content (Lan et al., 2022), suggesting that EPVS may contribute to abnormal ISF accumulation through specific pathological processes. It has been proposed that EPVS dysfunction may result in blood-brain barrier (BBB) disruption and neuroinflammation (Voorter et al., 2024; Lee et al., 2024). These changes may further increase the microvascular pressure and permeability of small arterioles and venules (Zhang et al., 2021), disrupting the perivascular fluid dynamics.

Deep medullary vein (DMV), integral components of the cerebral venous system, play a vital role in maintaining intracranial homeostasis, facilitating metabolic waste clearance, and regulating cerebrospinal fluid (CSF)-ISF exchange through interactions with the arterial system. Notably, the venous walls of DMV lack a smooth muscle layer (Lahna et al., 2022), rendering them highly susceptible to hemodynamic stress such as blood pressure variability and increased venous outflow resistance. These factors may lead to elevated venous pressure and altered permeability, thereby disturbing the stability of the perivascular microenvironment. Emerging evidence has identified a correlation between DMV dysfunction and increased cerebral ISF volume (Lan et al., 2024). On the basis of these observations, it is hypothesized that elevated EPVS score may contribute to DMV dysfunction, which in turn leads to increased ISF accumulation in brain tissue.

Accordingly, this study aimed to quantify extracellular ISF content using free water (FW) values derived from diffusion tensor imaging (DTI), evaluate EPVS severity via T2-weighted imaging (T2WI), and assess DMV function using susceptibility-weighted imaging (SWI)-based DMV score. The primary objective was to investigate the interrelationships between EPVS, DMV dysfunction, and ISF accumulation.

## 2 Materials and methods

### 2.1 Study population

This descriptive study prospectively enrolled 166 patients with CSVD consecutively recruited from the Department of Neurology between January and June 2025. The inclusion criteria were: (1) age > 40 years; (2) magnetic resonance imaging (MRI) findings consistent with the Standards for Reporting Vascular Changes on Neuroimaging (STRIVE) criteria for CSVD (Staals et al., 2014); (3) complete clinical data available; and (4) presence of at least one vascular risk factor. The exclusion criteria included: (1) severe intracranial arterial stenosis confirmed by ultrasound or computed tomography angiography; (2) acute cerebral infarction, intracerebral hemorrhage, or focal cerebral softening; (3) inflammatory, metabolic, or toxic demyelinating diseases; and (4) incomplete clinical or multimodal MRI data. The study was approved by the local ethics committee (Approval No. KY-2024058), and written informed consent was obtained from all participants. All procedures adhered to the principles of the Declaration of Helsinki.

Neuroimaging markers of CSVD include: White matter hyperintensity (WMH), Cerebral microbleeds (CMB), EPVS, and Lacunar infarcts (LI). WMH: Hyperintense lesions in periventricular or deep white matter on fluid-attenuated inversion recovery sequences. CMB: Homogeneous round hypointense foci (3–5 mm diameter) on SWI, excluding calcifications, vascular flow voids, and mineral deposits. EPVS: Small, punctate or linear hyperintensities along penetrating vessels on T2WI. LI: Ovoid lesions (3–15 mm diameter) showing hypointensity on T1WI and hyperintensity on T2WI. The CSVD score is defined as the sum of score from four neuroimaging markers of CSVD (0–4) (Staals et al., 2014).

### 2.2 Imaging protocol and parameters

All participants underwent multimodal MRI, including three-dimensional (3D) T1-weighted imaging (T1WI), T2WI, SWI, and DTI, performed using a 1.5 T scanner (MAGNETOM Aera; Siemens Medical Technologies, Munich, Germany). The acquisition parameters were as follows: 3D T1WI: repetition time (TR) = 2000 ms; echo time (TE) = 2.8 ms; slice thickness = 1 mm; interslice gap = 0.5 mm; field of view (FOV) = 23 × 23 cm^2^; matrix = 256 × 256. T2WI: TR = 3800 ms; TE = 88 ms; slice thickness = 5 mm; interslice gap = 1.5 mm; FOV = 23 × 23 cm^2^; matrix = 256 × 256. SWI: TR = 54 ms; TE = 40 ms; slice thickness = 3 mm; interslice gap = 1.5 mm; FOV = 23 × 23 cm^2^; matrix = 256 × 256. DTI: TR = 3600 ms; TE = 95 ms; slice thickness = 3 mm; interslice gap = 1.5 mm; FOV = 23 × 23 cm^2^; matrix = 128 × 128; diffusion directions = 30; *b*-values = 0, 1000, and 2000 s/mm^2^.

### 2.3 Clinical information

We collected baseline clinical data including age, sex, and vascular risk factors (hypertension, diabetes mellitus, hyperlipidemia, and smoking status). Hypertension was defined as systolic blood pressure > 140 mmHg or diastolic blood pressure > 90 mmHg on three consecutive occasions, or self-reported use of antihypertensive medication. Diabetes mellitus was defined as fasting plasma glucose > 11.1 mmol/L or self-reported use of antidiabetic medication. Hyperlipidemia was defined as a total cholesterol level > 6.2 mmol/L, a low-density lipoprotein cholesterol level > 4.14 mmol/L, high-density lipoprotein cholesterol < 1.04 mmol/L, triglycerides > 2.26 mmol/L, or use of lipid-lowering drugs. Smoking status counted current smokers.

### 2.4 EPVS scoring

**FIGURE 1:**
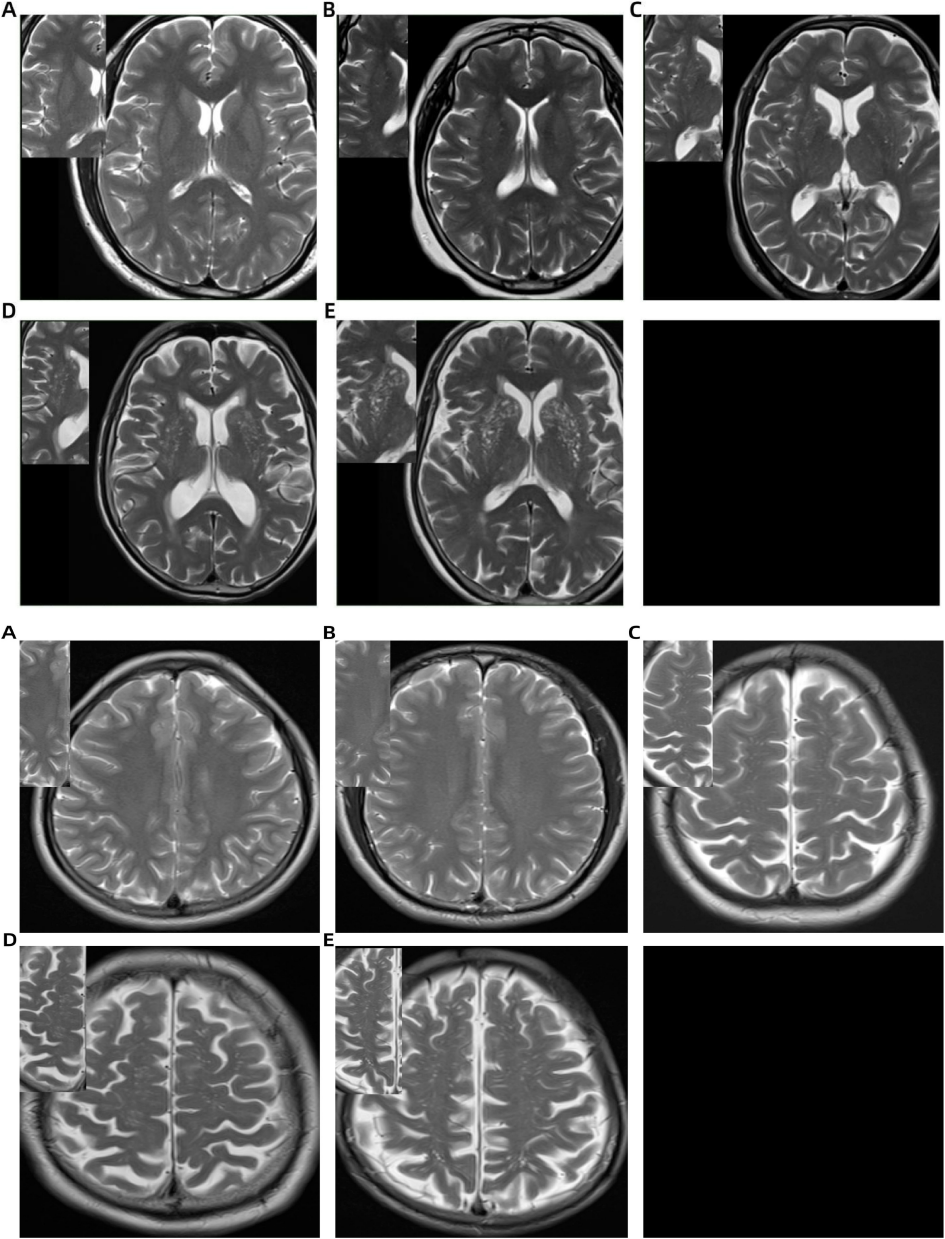
Basal ganglia (BG) and centrum semiovale (CSO) enlarged perivascular space (EPVS) scoring system on T2-weighted MRI: **(A)** no EPVS (score = 0); **(B)** 1–10 EPVS (score = 1); **(C)** 11–20 EPVS (score = 2); **(D)** 21–40 EPVS (score = 3); **(E)** >40 EPVS (score = 4).

EPVS were defined as small, punctate or linear hyperintensities on T2WI, typically measuring less than 3 mm in diameter and localized within the BG and CSO. The EPVS scoring assessment was autonomously conducted by two lead neuroimaging specialists. The Potter score was used to visually score the EPVS of both the BG and CSO sites separately (Potter et al., 2015). The level with the largest number of EPVS was selected, and if the left and right sides were asymmetrical, the side with more EPVS was selected. The scoring system was defined as follows: 0 = no EPVS; 1 = 1–10 EPVS; 2 = 11–20 EPVS; 3 = 21–40 EPVS; 4 ≥ 40 EPVS (Figure 1).

### 2.5 Measurement of DMV score

**FIGURE 2:**
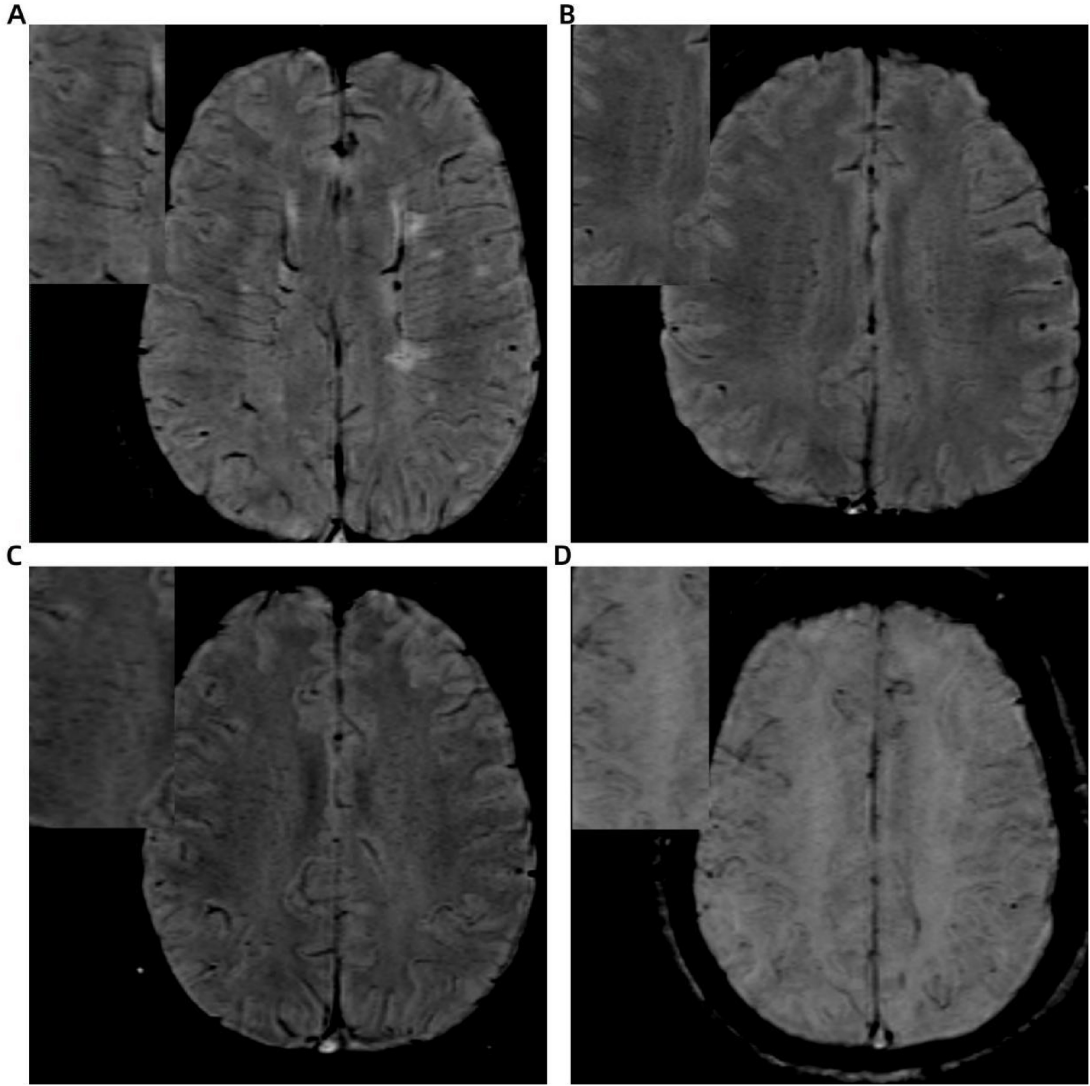
Illustration of deep medullary vein (DMV) scoring system based on SWI: **(A)** continuous, clear, and uniform DMV signal (score = 0); **(B)** continuous but heterogeneous signal (score = 1); **(C)** punctate or discontinuous weak signal (score = 2); **(D)** no visible DMV signal (score = 3).

The DMV scoring assessment was autonomously conducted by two lead neuroimaging specialists who were unaware of the medical and diagnostic data. The assessment was conducted using SWI images by selecting six DMV anatomical regions within the dual-sided anterior, central, and posterior regions near the side cavities, with each region scored according to DMV visibility. The DMV scoring criteria (Wang et al., 2023) were as follows: a score of 0 indicated uniformly continuous hypointense DMV signals; a score of 1 denoted clearly visible DMV signals with at least one interrupted venous continuity; a score of 2 represented punctate or extremely fine linear DMV signals; and a rating of 3 suggested total lack of observable DMV. The overall DMV rating was determined by adding up the ratings from all six areas, spanning 0 to 18 marks. The DMV score increases with greater vessel narrowing (Figure 2).

### 2.6 FW in white matter

**FIGURE 3:**
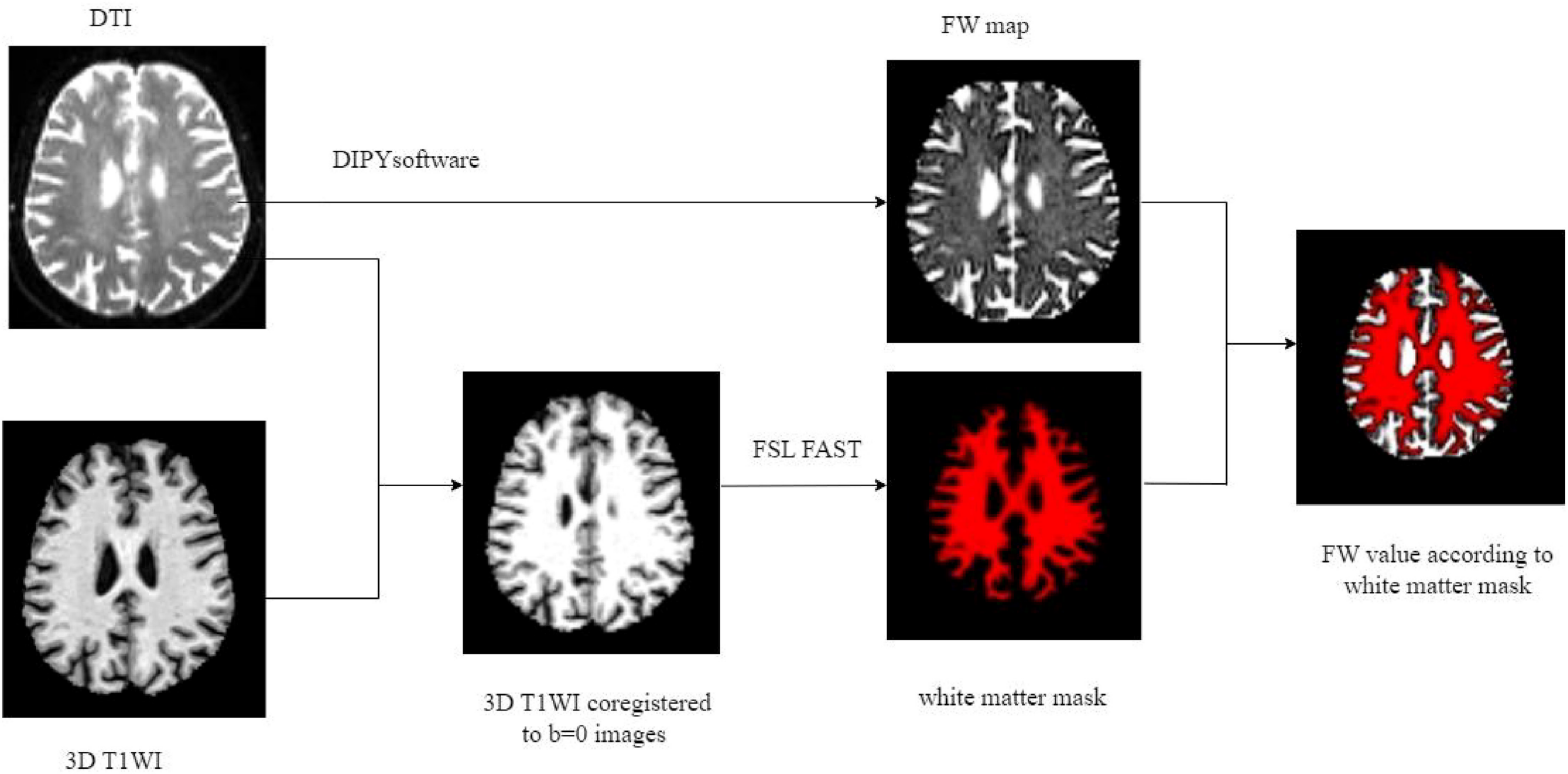
Illustration of FW processing. FW, free water; DTI, diffusion tensor imaging; T1WI, T1 weighted imaging; FSL FAST, DIPY post-processing tool.

The preprocessing of DTI data included noise reduction, Gibbs artifact removal, correction for gradient-echo distortions, and eddy current correction. Interstitial fluid (free water) distribution maps were then generated using a two-compartment model implemented in diffusion imaging in python (DIPY) software^1^ (Hoy et al., 2014). Briefly, in each voxel, the signal was fitted to a two compartment model, including a FW compartment (isotropic tensor) and a tissue compartment (FW-corrected tensor). The estimated parameters were the fractional volume of the FW compartment. Subsequent co-registration aligned the 3D T1WI with the *b* = 0 diffusion images. White matter FW values were extracted via tissue segmentation using the functional magnetic resonance imaging of the brain (FMRIB) Automated Segmentation Tool from the FMRIB Software Library (FSL) software suite, applied to the co-registered 3D-T1WI datasets (Figure 3). The fractional volume of the FW expresses the relative contribution of FW in each voxel, ranging from 0 to 1, with higher values indicating greater extracellular FW content.

### 2.7 Statistical analysis

Quantitative data with normal distribution were presented as mean ± standard deviation, while non-normally distributed variables were expressed as medians with interquartile ranges (IQR). Categorical variables were reported as frequencies and percentages. Associations between EPVS score, DMV score, and FW values were analyzed using Spearman’s rank correlation. A general linear model was applied to assess the factors influencing the FW values. To enhance the reliability of our findings, we constructed three hierarchical regression models: Model 1 (base model) with BG-EPVS as the independent variable and FW as the dependent variable; Model 2 (adjusted model), which extended Model 1 by including age, sex, and vascular risk factors (hypertension, hyperlipidemia, diabetes, and smoking status) as covariates to control for confounding effects; Model 3 (adjusted model), which further incorporated the DMV score into Model 2. The PROCESS for SPSS 2.16.3 framework [Model 4, a simple mediation effect model where an independent variable (predictor) influences a dependent variable (outcome variable) through a mediator]^2^ was also engaged to analyze the mediating effect of DMV score on BG-EPVS and FW. All statistical analyses were performed using SPSS version 27.0, and a two-tailed *p*-value < 0.05 was considered statistically significant.

## 3 Results

### 3.1 Baseline characteristics

A total of 166 patients were included, where the mean age was 63.6 ± 11.1 years. The imaging biomarker distributions were as follows: median BG-EPVS score was 1 (IQR: 1–2); median CSO-EPVS score was 1 (IQR: 1–2); CSVD score was 1 (IQR: 0–2), and median DMV score was 4 (IQR: 1–11). The mean FW values were 0.25 ± 0.02 (Table 1).

**TABLE 1:** Baseline characteristics of eligible study participants.

Variables	*n* = 166
Age, years	63.6 ± 11.1
Sex (m)	79 (47.6%)
Hypertension, yes	100 (60.2%)
Diabetes, yes	35 (21.1%)
Hyperlipidemia, yes	54 (32.5%)
Smoking status, yes	36 (21.7%)
BG-EPVS score	1 (1, 2)
CSO-EPVS score	1 (1, 2)
CSVD score	1 (0, 2)
DMV score	4 (1, 11)
FW	0.25 ± 0.02

BG-EPVS, basal ganglia enlarged perivascular space; CSO-EPVS, centrum semiovale enlarged perivascular space; CSVD, cerebral small vessel disease; DMV, deep medullary vein; FW, free water.

### 3.2 Inter-reader agreement for evaluation of BG-EPVS, CSO-EPVS and DMV score

The agreement between readers was excellent for the BG-EPVS score (Kappa = 0.94) and CSO-EPVS score (Kappa = 0.95), and the DMV score (Kappa = 0.92).

### 3.3 Relationship between BG-EPVS, CSO-EPVS, and FW

**FIGURE 4:**
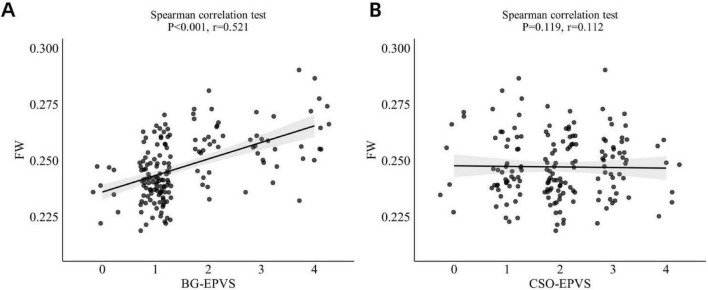
**(A)** Correlation between BG-EPVS and FW values. **(B)** Correlation between CSO-EPVS and FW values. BG-EPVS, basal ganglia enlarged perivascular space; CSO-EPVS, centrum semiovale enlarged perivascular space; FW, free water.

Spearman’s correlation analysis revealed a significant moderate positive correlation between BG-EPVS and FW (*r* = 0.521, *p* < 0.001; Figure 4A). In contrast, no significant association was found between CSO-EPVS and FW values (*r* = 0.112, *p* = 0.119; Figure 4B). A general linear model analysis (Model 1) confirmed an independent association between BG-EPVS and FW values (Table 2). This association remained statistically significant in Model 2 after adjustment for age, sex, and vascular risk factors (hypertension, hyperlipidemia, diabetes, and smoking status), but it lost significance in Model 3 after additional adjustment for the DMV score.

**TABLE 2:** Association between BG-EPVS and FW.

Variables	Model 1	*P*-value	Model 2	*P*-value	Model 3	*P*-value
	β (95% CI)		β (95% CI)		β (95% CI)	
Age, years			0.089 (0.056, 0.122)	<0.001	0.293 (0.101, 0.485)	0.003
Gender (m)			0.034 (−0.606, 0.674)	0.971	0.820 (−1.227, 2.866)	0.430
Hypertension, yes			0.046 (−0.559, 0.650)	0.882	1.343 (−0.830, 3.516)	0.224
Diabetes, yes			0.201 (−0.516, 0.918)	0.582	0.252 (−1.624, 2.127)	0.791
Hyperlipidemia, yes			0.004 (−0.620, 0.628)	0.991	0.692 (−1.916, 3.299)	0.601
Smoking status, yes			0.049 (−0.729, 0.828)	0.902	1.264 (−1.043, 3.571)	0.281
BG-EPVS score	1.155 (0.840, 1.470)	<0.001	0.721 (0.377, 1.066)	<0.001	0.131 (−0.037, 0.299)	0.125
DMV score					0.048 (0.014, 0.082)	0.006

Model 1: FW served as the primary outcome and BG-EPVS as the predictor. Model 2: Control for age, sex, hypertension, hyperlipidemia, diabetes, and smoking status. Model 3: Control for age, sex, hypertension, hyperlipidemia, diabetes, smoking status, and DMV score. BG-EPVS, basal ganglia enlarged perivascular space; FW, free water; DMV, deep medullary vein.

### 3.4 Relationship between BG-EPVS and DMV score

**FIGURE 5:**
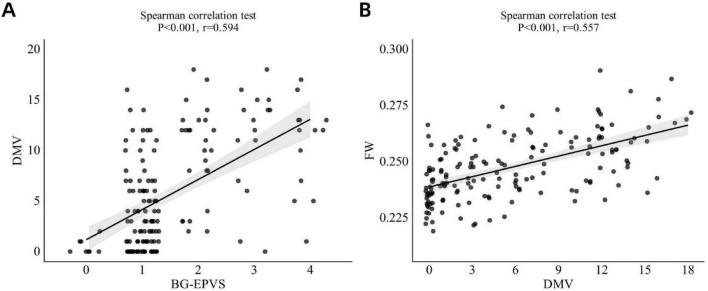
**(A)** Correlation betweenBG-EPVS and DMV score. **(B)** Correlation between DMV score and FW values. BG-EPVS, basal ganglia enlarged perivascular space; DMV, deep medullary vein; FW, free water.

Spearman’s correlation analysis showed a significant moderate positive correlation between BG-EPVS and DMV score (*r* = 0.594, *p* < 0.001; Figure 5A). Ordinal regression analysis (Model 1) indicated an independent association between BG-EPVS and DMV score (Table 3), which also remained statistically significant after controlling for age, sex, and vascular risk factors in Model 2.

**TABLE 3:** Association between BG-EPVS and DMV score.

Variables	Model 1	*P*-value	Model 2	*P*-value
	β (95% CI)		β (95% CI)	
Age, years			0.089 (0.056, 0.122)	<0.001
Sex (m)			0.034 (−0.606, 0.674)	0.971
Hypertension, yes			0.046 (−0.559, 0.650)	0.882
Diabetes, yes			0.201 (−0.516, 0.918)	0.582
Hyperlipidemia, yes			0.004 (−0.620, 0.628)	0.991
Smoking status, yes			0.049 (−0.729, 0.828)	0.902
BG-EPVS score	1.155 (0.840, 1.470)	<0.001	0.721 (0.377, 1.066)	<0.001

Model 1: DMV score served as the primary outcome and BG-EPVS as the predictor. Model 2: Control for age, sex, hypertension, hyperlipidemia, diabetes, and smoking status. BG-EPVS, basal ganglia enlarged perivascular space; DMV, deep medullary vein.

### 3.5 Relationship between DMV score and FW

Spearman’s correlation analysis indicated a significant moderate positive relationship between DMV score and FW (*r* = 0.557, *p* < 0.001; Figure 5B). The linear regression model (Model 1) revealed an autonomous link between DMV score and FW (Table 4). This relationship remained statistically significant after adjustment for age, sex, and vascular risk factors in Model 2.

**TABLE 4:** Association between DMV score and FW values.

Variables	Model 1	*P*-value	Model 2	*P*-value
	β (95% CI)		β (95% CI)	
Age, years			0.030 (0.013, 0.047)	<0.001
Sex (m)			−0.736 (−2.789, 1.318)	0.480
Hypertension, yes			1.475 (−0.702, 3.652)	0.183
Diabetes, yes			0.139 (−1.740, 2.018)	0.884
Hyperlipidemia, yes			−0.593 (−3.209, 2.024)	0.655
Smoking status, yes			−1.161 (−3.475, 1.154)	0.323
DMV score	0.100 (0.076, 0.125)	<0.001	0.057 (0.025, 0.089)	<0.001

Model 1: FW served as the primary outcome and DMV as the predictor. Model 2: Control for age, sex, hypertension, hyperlipidemia, diabetes, and smoking status. DMV, deep medullary vein; FW, free water.

### 3.6 Mediation analyses of the DMV score, BG-EPVS, and FW

**FIGURE 6:**
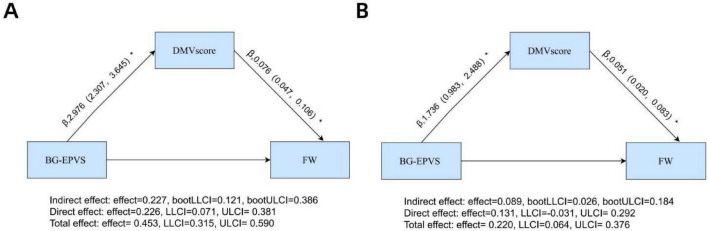
Mediation analyses of DMV score in the relationship between BG-EPVS and FW: **(A)** unadjusted model with BG-EPVS as predictor, DMV score as mediator, and FW as outcome; **(B)** model adjusted for age, sex, hypertension, hyperlipidemia, diabetes, and smoking status. *Statistical significance indicated by *p < 0.05. BG-EPVS, basal ganglia enlarged perivascular space; DMV, deep medullary vein; FW, free water.

The mediation analysis demonstrated a significant direct effect of BG-EPVS on FW (β = 0.226, 95% CI: 0.071–0.381, *P* < 0.05), with DMV score partially mediating the relationship between BG-EPVS and FW (β = 0.227, 95% CI: 0.121–0.386, *P* < 0.05; Figure 6A). This mediation accounted for 50.1% (0.227/0.453) of the total effect. After adjustment for age, sex, and vascular risk factors, the direct effect was no longer significant (β = 0.131, 95% CI: −0.031 to 0.292, *P* > 0.05), whereas the indirect effect remained significant (β = 0.089, 95% CI: 0.026–0.184, *P* < 0.05; Figure 6B). This mediation accounted for 40.4% (0.089/0.220) of the total effect.

## 4 Discussion

This study demonstrated a significant association between BG-EPVS and ISF content in the white matter, with DMV dysfunction acting as a partial mediator. Notably, this mediating effect remained statistically significant after adjusting for age, sex, and vascular risk factors. Specifically, higher BG-EPVS score was associated with more severe DMV impairment and greater extracellular ISF accumulation. These findings suggest a pathophysiological link between BG-EPVS, DMV dysfunction, and ISF homeostasis disruption.

Perivascular spaces are fluid-filled tubular structures that surround cerebral vessels and play a critical role in the clearance of metabolic waste via the CSF-ISF exchange system (Rasmussen et al., 2018). Aging and vascular risk factors contribute to progressive PVS dilation, resulting in EPVS, which impairs CSF-ISF circulation. In the BG, the primary drainage veins are the DMV. Pathologically, micro-arteriovenous pulsations surrounding the PVS serve as the driving force for perivascular clearance (Mestre et al., 2017). In response to EPVS dysfunction, these pulsations may be exaggerated as a compensatory mechanism.

DMV possess thin vascular walls and lack smooth muscle layers, rendering them particularly vulnerable to sustained hemodynamic stress. Chronic enhancement of pulsatile stress can lead to collagen deposition within venous walls, progressive venous stenosis, and reduced perfusion, resulting in interstitial edema and elevated ISF levels (Lahna et al., 2022). Over time, continued DMV narrowing may further increase venous pressure, promote vascular wall permeability, and exacerbate leakage into the interstitial compartment, thereby amplifying ISF accumulation. Additionally, compensatory increases in arterial pulsatility may propagate into adjacent venous structures, including the DMV, further contributing to ISF elevation (Fulop et al., 2019).

Furthermore, dysfunction of the CSF-ISF circulation can compromise the integrity of the BBB (Voorter et al., 2024), increasing the permeability of DMV vessel walls, which exacerbates vascular leakage and results in vasogenic edema and elevated ISF volume. In parallel, impaired CSF-ISF exchange facilitates the accumulation of neurotoxic metabolic byproducts (Duering et al., 2018), which can trigger neuroinflammation. In turn, neuroinflammatory processes further disrupt BBB integrity, enhancing vascular permeability and promoting additional ISF accumulation. Moreover, neuroinflammation may induce perivascular space fibrosis, thereby contributing to the formation and persistence of EPVS (Brown et al., 2018), further worsening CSF-ISF circulatory dysfunction and establishing a self-perpetuating pathological cycle.

In this study, a significant moderate positive correlation was observed between BG-EPVS score and FW values, whereas no such correlation was found for CSO-EPVS score. This disparity may reflect region-specific differences in the underlying pathophysiological mechanisms. BG-EPVS is consistently associated with hypertension (Klarenbeek et al., 2013). Elevated arterial pressure and increased pulsatility can compromise DMV function in the BG, resulting in impaired venous drainage and elevated ISF levels. Additionally, intensified vascular pulsations can render penetrating arterioles in the BG more vulnerable to damage, facilitating the development of EPVS (Rivera-Rivera et al., 2017) and aggravating ISF accumulation. In contrast, CSO-EPVS is more frequently linked to cerebral amyloid angiopathy (Banerjee et al., 2018). Histopathological studies have shown that amyloid β-protein preferentially deposits around cortical and leptomeningeal arteries, with relatively minimal involvement of BG vasculature (Jäkel et al., 2022). Furthermore, amyloid deposition primarily affects arterial vessels rather than veins, and the CSO is drained by multiple cortical and superficial medullary veins, which may permit collateral compensation in the event of focal venous dysfunction. Additionally, compared to the BG nuclei, the white matter fiber tracts in the CSO exhibit lower metabolic activity. Consequently, BBB disruption and neuroinflammatory responses triggered by ischemic-hypoxic insults are likely milder in this region, resulting in a reduced impact on interstitial fluid retention. Furthermore, even in cases of severe EPVS within the CSO, the lesions exhibit spatially discrete distribution. This spatial characteristic may mitigate the impact of CSO-EPVS on interstitial fluid through compensatory mechanisms of adjacent normal brain regions.

Moreover, our study revealed a significant direct effect between BG-EPVS and FW. These indicates that BG-EPVS may impair the CSF-ISF circulation, leading to the retention of ISF in the periventricular white matter and resulting in increased FW values. On the other hand, dysfunction in CSF-ISF exchange hinders the effective clearance of harmful metabolic substances, which may trigger neuroinflammation and further elevate FW values. Moreover, this suggest the potential involvement of other unmeasured mediating pathways (e.g., age, vascular risk factors, BBB disruption, neuroinflammation) in FW development. After adjusting for age, sex, and vascular risk factors, DMV completely mediated the effect of BG-EPVS on FW. This further verifies the robustness of DMV as a mediating factor and demonstrates the reliability of our findings.

Several limitations should be acknowledged. First, this was a single-center study with a relatively small sample size, which may have introduced selection bias and limited generalizability. Future multicenter studies with larger cohorts are needed to validate and extend these findings. Second, despite employing a methodologically rigorous design with robust data quality, and statistical evidence supporting the mediating role of DMV in the BG-EPVS and FW. But, the cross-sectional nature of this study precluded the evaluation of temporal changes in BG-EPVS, DMV score, and FW values. So, the directionality of the mediation pathway should be interpreted with caution. Longitudinal studies are therefore essential to elucidate the progression of these imaging biomarkers over time. Third, direct quantification of BBB integrity and neuroinflammatory biomarkers was not performed; these unmeasured factors may constitute residual confounders in the mediation analysis. Finally, EPVS scoring in this study was based on a semi-quantitative visual scale. Automated volumetric assessment using dedicated software may provide a more precise and objective measure of EPVS burden and progression in future research.

In summary, higher BG-EPVS score is independently associated with increased extracellular ISF content in the white matter, and DMV dysfunction is a key mediator of this relationship.

## Data Availability

The data analyzed in this study is subject to the following licenses/restrictions: if anyone is interested in our data, please contact the corresponding author of this study. Requests to access these datasets should be directed to lkw18957091019@163.com.

## Footnotes

1
https://dipy.org

2
https://afhayes.com
